# PERK Regulates the Proliferation and Development of Insulin-Secreting Beta-Cell Tumors in the Endocrine Pancreas of Mice

**DOI:** 10.1371/journal.pone.0008008

**Published:** 2009-11-24

**Authors:** Sounak Gupta, Barbara McGrath, Douglas R. Cavener

**Affiliations:** 1 Department of Biology, Pennsylvania State University, University Park, Pennsylvania, United States of America; 2 The Huck Institutes of the Life Sciences, Pennsylvania State University, University Park, Pennsylvania, United States of America; Roswell Park Cancer Institute, United States of America

## Abstract

**Background:**

PERK eIF2α kinase is required for the proliferation of the insulin-secreting beta- cells as well as insulin synthesis and secretion. In addition, PERK signaling has been found to be an important factor in determining growth and angiogenesis of specific types of tumors, and was attributed to PERK-dependent regulation of the hypoxic stress response. In this report we examine the role of PERK in regulating proliferation and angiogenesis of transformed beta-cells in the development of insulinomas.

**Methodology:**

The SV40 Large T-antigen (Tag) was genetically introduced into the insulin secreting beta-cells of *Perk KO* mice under the control of an inducible promoter. Tumor growth and the related parameters of cell proliferation were measured. In late stage insulinomas the degree of vascularity was determined.

**Principal Findings:**

The formation and growth of insulinomas in *Perk*-deficient mice was dramatically ablated with much fewer tumors, which averaged 38-fold smaller than seen in wild-type control mice. Beta-cell proliferation was ablated in *Perk*-deficient mice associated with reduced tumor growth. In the small number of large encapsulated insulinomas that developed in *Perk*-deficient mice, we found a dramatic reduction in tumor vascularity compared to similar sized insulinomas in wild-type mice. Although insulinoma growth in *Perk*-deficient mice was largely impaired, beta-cell mass was increased sufficiently by T-antigen induction to rescue the hypoinsulinemia and diabetes in these mice.

**Conclusions:**

We conclude that PERK has two roles in the development of beta-cell insulinomas, first to support rapid cell proliferation during the initial transition to islet hyperplasia and later to promote angiogenesis during the progression to late-stage encapsulated tumors.

## Introduction

Tumor growth is dependent upon high rates of protein synthesis, and previous studies had shown that control of protein synthesis mediated by phosphorylation of the translation initiation factor eIF2α is important for tumor progression [1], [2], [3], [4]. Humans and other mammals have four eIF2α kinases including GCN2, HRI, PKR and PERK. Previous studies have shown that tumors that lack PERK-mediated signaling tend to be smaller and this was found to be correlated with smaller hypoxic microenvironments [5], [6], [7]. In these studies immortalized *Perk*-deficient MEFs transformed with oncogenic Ki-RasV12 or HT29 colorectal carcinoma cells stably expressing a dominant negative *Perk* allele [8] were transplanted into nude mice [6], [7]. The resultant tumors were found to be smaller and less vascular compared to control implants that were wild type for *Perk* [6], [7]. However, in these studies, the dramatic reduction in growth of the *Perk*-deficient tumors was attributed to increased rates of apoptotic cell death and impaired angiogenesis but, inexplicably, tumor cell proliferation rates were not examined [6], [7].

*Perk* deficiency in humans is the cause of the Wolcott-Rallison Syndrome characterized by permanent neonatal diabetes, exocrine pancreas deficiency, osteopenia, and growth retardation [9] and these defects are recapitulated in *Perk* KO mice [10], [11], [12]. We discovered that the diabetes in *Perk KO* mice was due to hypoinsulinemia associated with low insulin-secreting beta-cell mass caused by diminished beta-cell proliferation and impaired insulin secretion [13]. Gene expression analyses revealed reduced expression of factors vital to the G2-M cell cycle transition [13]. Moreover, *Perk*-deficient osteoblasts exhibited reduced levels of cell proliferation [14]. Having established the importance of PERK in beta-cell proliferation we decided to investigate whether PERK played an important role in the progression of insulinomas, a pancreatic beta-cell cancer.

## Results

### Growth of Insulinomas Induced by the SV40 Large T-Antigen Is Severely Blunted in PERK-Deficient Mice

**Figure 1:**
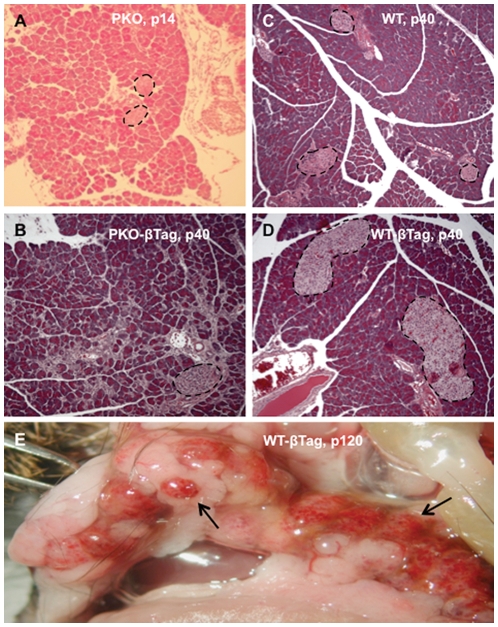
The growth of beta-cell insulinomas is ablated in *Perk*-deficient mice. (**A**–**B**) Pancreata of *wildtype* (*WT*) mice with (**A**) or without (**B**) beta-cell specific SV40 T-antigen (*βTag*) were examined histologically after hematoxlin and eosin staining to assess relative beta-cell hyperplasia at postnatal day 40 (p40). (**C**) Representative necropsy image of the whole pancreata of a p120 *WT-βTag* mouse showing multiple insulinomas dispersed throughout the pancreas (arrows). (**D**) *Perk KO* (*PKO*) mice displayed relatively small islets at p14. (**E**) *PKO-βTag* mice exhibited increased beta cell mass.

To generate insulinomas in mice, the SV40 Large T-Antigen was introduced using a bipartite genetic system comprised of the *tet-Tag* transgene and the *RIP7-rtTA* transgene. Together these transgenes (denoted *βTag*) provide beta-cell specific, doxycycline-inducible control of the expression of the T-antigen [15]. These strains were further crossed into *Perk KO* (*PKO*) mice to study the effect of *Perk* on postnatal β-cell proliferation. As reported previously, we found that the expression of *βTag* in wild-type (*WT-βTag*) mice caused islet hyperplasia by postnatal day 40 (p40) (Figure 1A–B), and these hyperplastic islets progressed to highly vascular insulinomas associated with hyperinsulinemia and hypoglycemia (arrows, Figure 1C) [15]. Serum insulin increased by >40-fold compared to normal in some animals (not shown).

**Figure 2:**
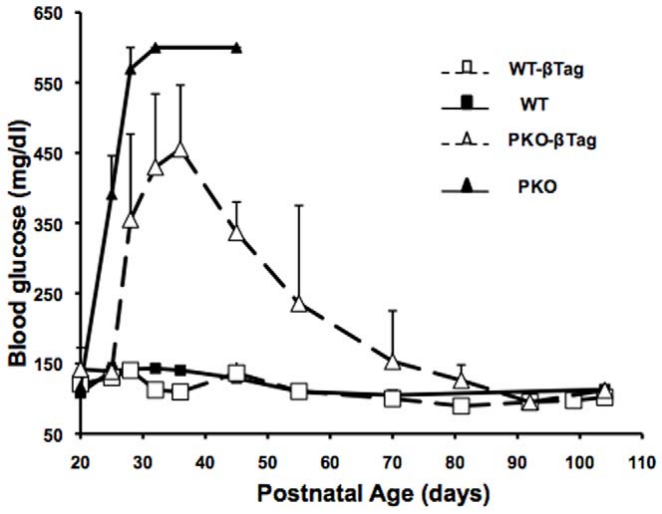
Beta-cell hyperplasia caused by T-antigen transformation reverses diabetes in *Perk KO* mice. Serum glucose levels (mg/dl) in wildtype (*WT*, closed squares and solid line), *wildtype-βTag* (*WT-βTag*, open squares and dashed line), *Perk KO* (*PKO*, closed triangles and solid line) and *Perk KO-βTag* (*PKO-βTag*, open triangles and dashed line) littermates during postnatal progression of insulinoma development. Each data point is the mean of 4 to 13 mice. Serum glucose in *PKO* mice often exceeded the detection capability of the blood glucose monitor (600 mg/dl) and these mice didn't survive beyond p45. In this figure and all others, error bars = SEM.

**Figure 3:**
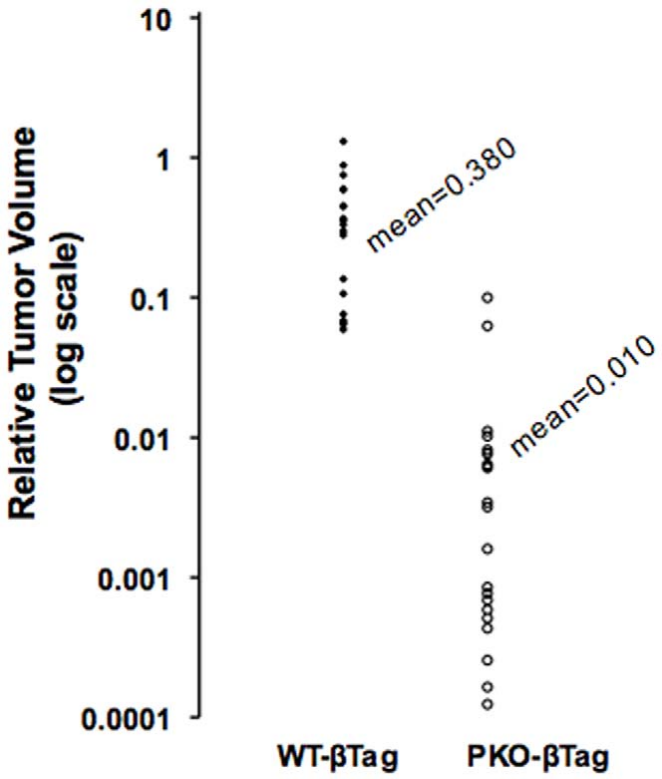
Relative tumor volumes in *βTag* induced insulinomas in wild type and *Perk KO* littermates. The insulinomas exhibited a nearly spherical shape and therefore tumor volumes were estimated as a function of their diameter. The tumor volumes of the largest insulinomas from three *PKO-βTag* mice (n = 23) and 6 *WT-βTag* mice (n = 19) at age p120 are plotted on a logarithmic scale showing a 38-fold difference. *****P<0.000005.

In the absence of *βTag*, *Perk*-deficient mice have a low beta-cell mass at postnatal day p14 (Figure 1D) and progress to overt diabetes by p21 [13]. Most *Perk KO* mice die due to numerous complications by 5 to 6 weeks of life [12]. Introduction of *βTag* into *Perk KO* mice (*PKO-βTag*) resulted in partial rescue of the beta-cell mass in p40 juvenile mice (Figure 1E) and delayed the onset of overt diabetes by one week. Remarkably, continued islet growth and increased beta-cell mass resulted in complete reversal of the diabetes by 7–10 weeks (Figure 2). Blood glucose levels in *PKO-βTag* mice were found to peak by p35-p40, followed by a gradual progression to hypoglycemia (Figure 2). Although T-antigen expression was able to induce beta-cell proliferation and reverse diabetes in the *Perk KO* mouse, the size of their insulinomas were on average 38-fold smaller than seen in wild-type littermates (Figure 3).

### Beta-Cell Death Does Not Explain the Reduced Beta-Cell Mass in *PKO-βTag* Mice

**Figure 4:**
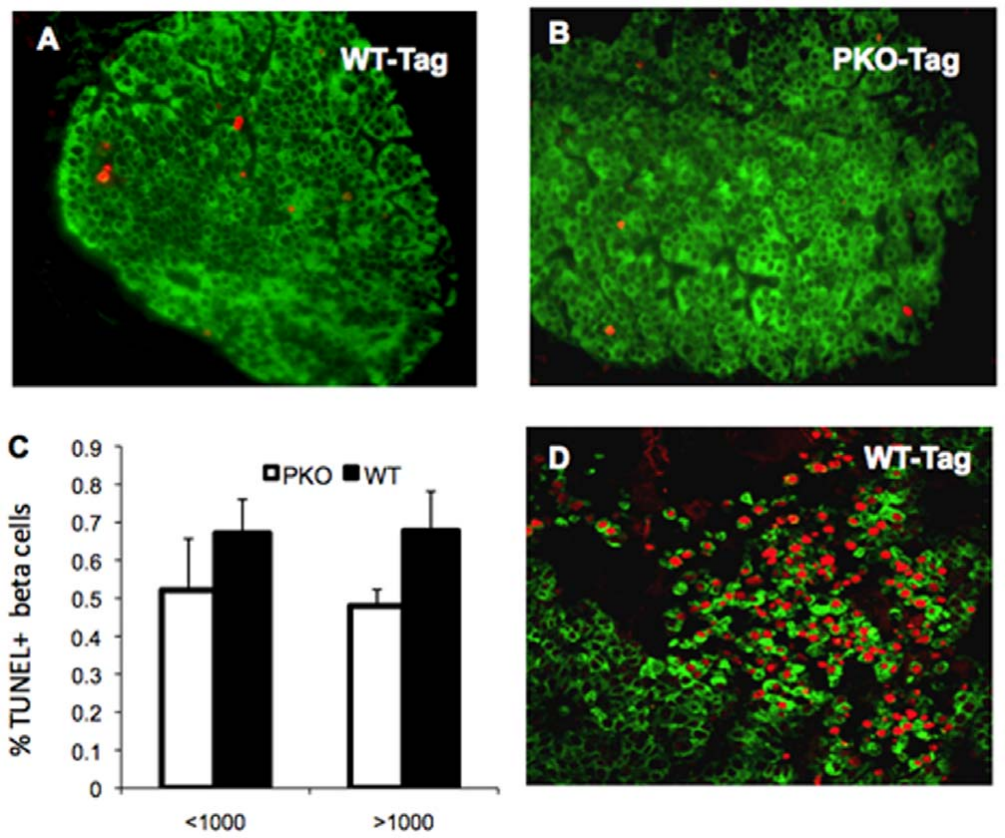
Beta-cell death does not significantly contribute to tumor size differences between *Perk* genotypes. TUNEL positive beta-cells were detected by immunohistochemistry. (TUNEL = red, insulin = green). (**A**) *WT-βTag* islet p120, 40X, (**B**) *PKO-βTag* islet p120, 40X, (**C**) Beta-cell death was estimated as the percentage of beta-cells (insulin positive) that exhibited a high level of DNA fragmentation as detected by TUNEL antibody in the nucleus. Islets were categorized into two groups: smaller than 1,000 beta-cells and larger than 1,000 beta-cells counted on a single cross-sectional area of each islet. The mean and standard error are shown for TUNEL positive beta-cells for each islet size class. No significant differences were seen between genotypes for either size class. For the small islet size class, *WT-βTag* n = 27 islets (12,946 total beta-cells) and *PKO-βTag* n = 20 islets (4,749 total beta-cells). For the large islet size class, *WT-βTag* mice n = 20 islets (138,858 total beta-cells) and *PKO-βTag* n = 12 islets (59,109 total beta-cells). (**D**) A small section of a very large beta-cell insulinoma from a p120 *WT-βTag* mouse showing a high density of TUNEL positive beta-cells (40X). Four other distinct clusters of beta-cells also showed very high percentage of beta-cells that were TUNEL positive in this cross section of the tumor (not shown), whereas the majority of tumor exhibited a frequency of less than 1% TUNEL positive beta-cells. The TUNEL positive beta-cell clusters tend to be centrally located in the tumor.

To determine if differential cell death may be the cause for the relatively small size of the insulinoma in *PKO-βTag* mice, we measured the percentage of beta-cells exhibiting TUNEL positive nuclei in *PKO-βTag* and *WT-βTag* mice during the exponential growth phase of the developing beta-cell tumors. Examination of islets smaller or larger than 1,000 beta-cells revealed that the percentage of TUNEL positive beta-cells was modestly higher in *WT-βTag* mice compared to *PKO-βTag* mice for both islet size classes (Figure 4A–C) but did not reach statistical significance. In one *WT-βTag* mouse we examined an extraordinarily large tumor and found dense clusters of TUNEL positive beta-cells (Figure 4D). Within the local region of these clusters >10% of the beta-cells were TUNEL positive whereas the majority of the tumor exhibited a frequency of less than 1% similar to the other tumors in *WT-βTag* mice. The data from this exceptional tumor were not included in Figure 4C. Although beta-cell death may contribute to the tumor size difference between *WT-βTag* mice and *PKO-βTag* mice these data suggest that cell death differences would tend to lessen the difference between these genotypes.

### Beta-Cell Proliferation in *PKO-βTag* Mice Is Reduced Compared to *WT-βTag* Mice

The rates of beta-cell proliferation in *PKO-βTag* mice and age and sex matched *WT-βTag* littermates were estimated by BrdU incorporation. As larger neoplastic islets have higher rates of proliferation, beta-cell proliferation rates were assessed in uniform islets of less than 50 cells in *PKO-βTag* and their *WT-βTag* littermates at the following postnatal ages: p50, p100, and p120. *PKO-βTag* islets showed a statistically significant 2.2 fold reduction in beta-cell proliferation (Table 1). Furthermore, rates of beta-cell proliferation were found to be consistently reduced in *PKO-βTag* mice both in the early phases of *βTag* induced islet hyperplasia at p50 (3.1 fold difference) as well as in the later stages of the progression to insulinomas at p120 (1.6 fold difference) (Table 1).

**Table 1:** Beta-cell proliferation is impaired in *Perk*-deficient, T-antigen transformed islets.

Genotype	Postnatal Age	Analysis Criteria	Beta-cells/islet	% BrdU+ cells
WT-βTag	p50, n = 1	all islets, n = 7	204.7	5.6
PKO-βTag	p50, n = 1	all islets, n = 8	40.3	1.8
				P<0.05
WT-βTag	p50/100/120, n = 3	islets<50 cells, n = 28	39.6	4.6
PKO-βTag	p50/100/120, n = 3	islets<50 cells, n = 25	21.6	2.1
				P<0.05
WT-βTag	p120, n = 1	islets>500 cells, n = 9	3869.2	8.0
PKO-βTag	p120, n = 1	islets>500 cells, n = 11	2115.2	5.1
				P<0.05

Beta-cell proliferation rates were assessed by BrdU incorporation in age and sex matched *PKO-βTag* mice and *WT-βTag* littermates at postnatal ages p50, p100 and p120. Cells = insulin-positive beta-cells.

Although the expression of the T-antigen in beta-cells leads to a dramatic increase in beta-cell proliferation in adult mice, we found that beta-cell masses at embryonic day 16.5 were not significantly different either between wild-type mice with or without *βTag* expression as well as their *PKO-βTag* littermates (not shown). Our findings that *βTag* expression did not significantly affect beta-cell mass in wild-type and *Perk*-deficient mice in embryonic mice (e16.5) is consistent with previous reports that have shown that *βTag* promotes postnatal beta-cell proliferation beginning at about 3 weeks [15]. Furthermore, Tag is an oncogene that functions primarily by inactivating the retinoblastoma protein (the main gate-keeper for the G1-S cell cycle checkpoint) and p53 (an inducer of apoptosis) [15], and previous studies have shown that the negative regulation of the G1-S cell cycle checkpoint does not significantly affect late-embryonic beta-cell neogenesis [16]. Our lab has also shown that beta-cell mass in *Perk*-deficient mice is comparable to their wild-type littermates in the early embryonic stages following neogenesis of beta-cells from endocrine progenitor cells [13].

### T-Antigen Induced Insulinoma Vascularity Is Greatly Diminished in *Perk*-Deficient Mice

**Figure 5:**
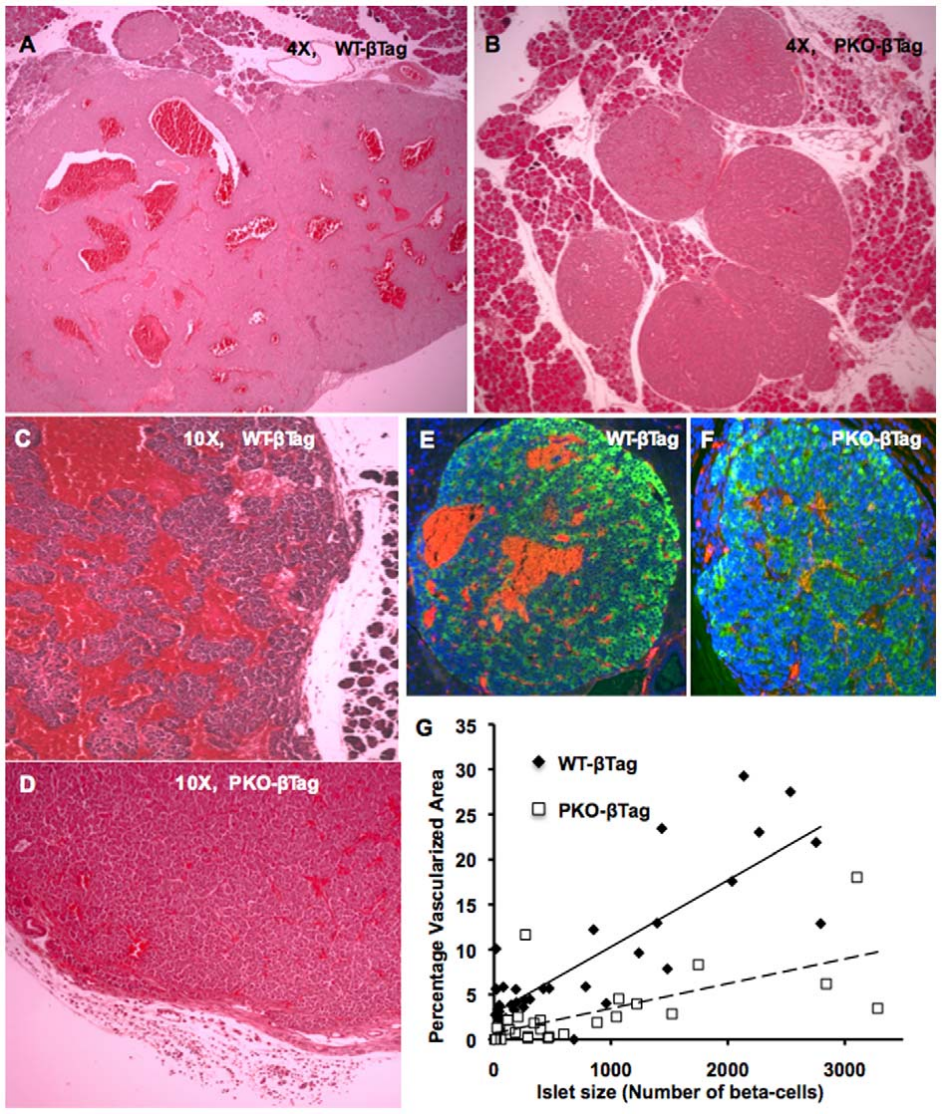
*Perk*-deficient insulinomas exhibit dramatically reduced vascularity. (**A**–**D**)**:** Insulinomas from p120 *WT-βTag* (**A, C**) and *PKO-βTag* (**B, D**) mice stained with hematoxylin and eosin. Insulinomas from p120 *WT-βTag* (**E**) and *PKO-βTag* (**F**) littermates were stained for insulin (green), BrdU (red) and DAPI (blue). (**E**) Note the red autofluorescent signal from red blood cells within the highly vascularized *WT-βTag* insulinomas. (**G**) The vascularized area in the cross sectional images of the islets were expressed as a percentage of the total islet area in *PKO-βTag* mice and *WT-βTag* littermates at p120. For each islet, the percentage vascularized area was plotted on the y-axis and the corresponding islet size was plotted on the x-axis. *WT-βTag:* n = 2 mice, number of islets analyzed = 37, solid line represents the linear trend, average islet size = 1367.2, and the percent vascularized area = 8.9%. *PKO-βTag*: n = 2 mice, number of islets analyzed = 30, dashed line represents the linear trend, average islet size = 898.3, and the percentage vascularized area = 2.9%. The difference in the average value of the vascularized area between *PKO-βTag* and *WT-βTag* was highly significant, ***P-value<0.0005.

T-antigen induced beta-cell proliferation eventually leads to the formation of insulinomas [15], [17]. These insulinomas exhibited a marked increase in vascularity by 11–14 weeks (Figure 1E) compared to the initial stages of islet hyperplasia (4–6 weeks). Histological examination of *WT-βTag* insulinomas at p120 revealed highly vascularized tumors (Fig. 5A, C, E). In contrast, insulinomas from *PKO-βTag* littermates appeared to have significantly reduced tumor vascularity (Figure 5B). Because *PKO-βTag* tumors are generally much smaller, their lack of vascularity could be simply due to a failure to progress to late stage insulinoma. To address this issue, we compared size-matched insulinomas from *Perk KO* and wild-type mice, and found a statistically significant 3-fold decrease in tumor vascularity in *PKO-βTag* insulinomas (Figure 5G). Moreover, in the small number of very large encapsulated insulinomas in *PKO-βTag* mice, we found a dramatic reduction in tumor vascularity (Figure 5D) compared to size-matched tumors in *WT-βTag* mice (Figure 5C).

## Discussion

Previous studies have identified three distinct stages of T-antigen-induced beta-cell tumor progression including islet hyperplasia, islet-angiogenesis and finally formation of solid encapsulated tumors [18], [19]. The predominant determinant in the progression of normal islets to a stage of hyperplasia and angiogenesis was found to be the rate of beta-cell proliferation, while reduced apoptosis was critical in the progression to solid encapsulated tumors [18], [19]. As expected, we observed a similar progression to insulinomas and associated hyperinsulinemia-hypoglycemia in *wildtype-βTag* mice. However, beta-cell proliferation was significantly reduced in *Perk-*deficient *βTag* mice and this defect was most prominent in the initial transition to islet hyperplasia. Insulinomas in *Perk KO-βTag* mice were fewer, the average size was 38-fold smaller, and they rarely develop into encapsulated tumors. Cell death was slightly higher in *wildtype-βTag* mice and therefore does not appear to contribute to the smaller size of tumors in *Perk KO-βTag* mice. Beta-cells isolated from *Perk KO-βTag* insulinomas also showed poor growth characteristics in culture, and we were unable to establish a T-antigen, *Perk*-deficient beta-cell line from isolated insulinomas from these mice whereas we could readily establish them from the *wildtype-βTag* mice. Although the T-antigen induction of beta-cell proliferation in *Perk KO* was blunted compared to the wild-type mice, the increase in beta-cell mass was nonetheless sufficient to eventually reverse the diabetes of these mice.

During the later stages of tumor development hypoxia becomes more prevalent as cell growth outstrips the pre-existing vascular system [5], [20]. To provide increased circulation within the tumor microenvironment, angiogenesis is induced in part by the response to hypoxia [21]. PERK has been shown to be important for regulating the hypoxic stress response in fibroblast-derived tumors [6], [7] and it was speculated that the slow growth of *Perk*-deficient tumors was caused by a defect in the hypoxia-stress response resulting in increased cell death. We found that *Perk*-deficient insulinomas failed to develop extensive vasculature, consistent with the hypothesis that PERK plays a critical role in regulating the response to hypoxia. However, we discovered that beta-cell proliferation and beta-cell mass in *Perk KO-βTag* islets substantially lagged behind *wildtype-βTag* islets during the initial hyperplasia stage prior to the time that hypoxic conditions exist. Moreover, beta-cell death was actually slightly higher in the islets of the wildtype*-βTag* mice during the exponential growth phase and therefore does not contribute to the relatively small size of the *Perk KO-βTag* islets. Thus we suggest that PERK has two roles in tumor formation, first to support rapid cell proliferation and then later to promote angiogenesis. In the absence of PERK, however, tumors may not achieve sufficient size in order for severe hypoxia and cell death to occur. We found high apparent beta-cell death only in a very large *wildtype-βTag* insulinoma, but similarly sized insulinomas were never seen in *Perk KO-βTag* mice. To examine PERK's role in late stage tumor progression it will be necessary to acutely ablate PERK expression later in tumor development.

In addition to regulating beta-cell functions that contribute tumor progression, PERK may also regulate functions in other cell types including the vascular endothelial cells that comprise the tumor macroenvironment. In our studies *Perk* is deficient in all cells of the entire animal, and therefore we cannot discriminate between PERK's functions in these different cell types. We are currently generating Tag-induced insulinomas in mice in which *Perk* has been specifically ablated in various cell types of the pancreas to determine the role of PERK in both the beta-cells and macroenvironment of the developing tumor.

The molecular mechanism underlying PERK-dependent regulation of tumor growth and angiogenesis is unknown. We speculate that reduced proliferation and vascularity in *Perk*-deficient insulinomas is caused by a fundamental defect in function in the secretory pathway that we have recently discovered in beta-cells. *Perk*-deficient beta-cells have defects in [13] ER to Golgi trafficking, ER associated proteasomal degradation, and integrity of the ER and Golgi (unpublished). Cell proliferation and membrane targeting of angiogenic receptors (e.g. VEGFRs) are highly dependent upon normal ER functions. We therefore suggest that these defects in ER functions may negatively impact beta-cell proliferation and angiogenesis.

### Ethics

All the procedures that involved animal subjects were approved by the IACUC of the Pennsylvania State University in accordance with federal and state regulations governing care and use of animals.

## Materials and Methods

### Genetic Strains

Mice expressing SV40 large T-antigen conditionally in beta-cells were generated by crossing heterozygous *tet-Tag* transgenic mice with heterozygous *RIP7-rtTA* transgenic mice [15]. To activate Tag expression, double-transgenic mice (denoted as *βTag*) were treated with 2 mg/ml doxycycline (Sigma) (in 2.5% sucrose) in their drinking water. Doxycycline treatment was administered in utero and continued postnatally. Untreated double-transgenic mice or doxycycline-treated mice lacking one of the two transgenes served as controls. These mice were further crossed into *Perk* ^+/−^ strains [12] to generate *PKO-βTag* and *WT-βTag* mice. All the strains were in a mixed genetic background. All the procedures that involved animal subjects were approved by the IACUC of the Pennsylvania State University.

### Blood Glucose Measurements

Blood samples were obtained from the tail and were measured for glucose concentration using the Accu-Chek or OneTouch Ultra glucometers.

### Histological Analyses and Immunohistochemistry

After the animals were sacrificed by CO_2_ asphyxiation, the pancreata were removed, fixed in 4% paraformaldehyde, dehydrated, paraffin-embedded and sectioned. Sections were processed for immunohistochemistry or stained with hematoxylin and eosin (H & E) by standard procedures. Briefly, cells were permeabilized in 0.2% Triton-X in phosphate-buffered saline (PBS) for 10 minutes. In the case of BrdU, antigen retrieval was performed by boiling in citric buffer for 10 mins. This was followed by serial incubations with the appropriate primary and secondary antibodies. The following antibodies were used: insulin (Linco Research), glucagon (Santa Cruz), proinsulin (Beta-Cell Biology Consortium), and BrdU (DAKO). Appropriate secondary antibodies conjugated with Alexa Fluor 488 or 555 dye (Molecular Probes) were used to visualize the labels. DAPI was used for nuclear staining. Fluorescence images were captured and analyzed with a Nikon Eclipse E1000 microscope and Image-Pro Plus Software (Phase 3 Imaging Systems).

### Beta-Cell Mass and Proliferation Analysis

Mice were injected intraperitoneally with 100 µg BrdU/gram body weight. The pancreata were removed 6 hrs later, and processed. For the estimation of beta-cell mass, embryonic pancreata were processed and all of the islets in a total of 6 tissue sections (covering the entire pancreas) were analyzed.

### Statistical Analysis

Statistical analysis was performed using Student's t-test; *P*<0.05 was accepted as significant. Error bars represent the standard error of mean (SEM).
