# Correction: PERK Regulates the Proliferation and Development of Insulin-Secreting Beta-Cell Tumors in the Endocrine Pancreas of Mice

**DOI:** 10.1371/annotation/b22a2657-7ce6-471a-8593-8bc8e86e2efa

**Published:** 2009-12-11

**Authors:** Sounak Gupta, Barbara McGrath, Douglas R. Cavener

Figure 1 is incorrect. Please view the correct figure here:

**Figure 1:**
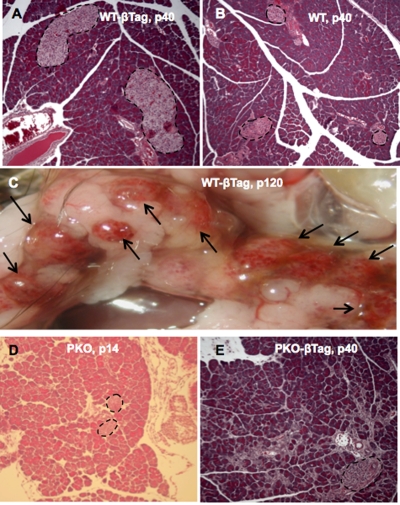
The growth of beta-cell insulinomas is ablated in *Perk*-deficient mice.

